# Negative regulation of amino acid signaling by MAPK-regulated 4F2hc/Girdin complex

**DOI:** 10.1371/journal.pbio.2005090

**Published:** 2018-03-14

**Authors:** Liang Weng, Yi-Peng Han, Atsushi Enomoto, Yasuyuki Kitaura, Shushi Nagamori, Yoshikatsu Kanai, Naoya Asai, Jian An, Maki Takagishi, Masato Asai, Shinji Mii, Takashi Masuko, Yoshiharu Shimomura, Masahide Takahashi

**Affiliations:** 1 Department of Pathology, Nagoya University Graduate School of Medicine, Nagoya, Japan; 2 Department of Applied Molecular Biosciences, Graduate School of Bioagricultural Sciences, Nagoya University, Nagoya, Japan; 3 Laboratory of Biomolecular Dynamics, Department of Collaborative Research, Nara Medical University, Kashihara, Nara, Japan; 4 Department of Bio-System Pharmacology, Graduate School of Medicine, Osaka University, Suita, Osaka, Japan; 5 Department of Respiratory Medicine, Xiangya Hospital, Central South University, Kaifu District, Changsha, China; 6 Cell Biology Laboratory, Department of Pharmaceutical Sciences, Faculty of Pharmacy, Kindai University, Higashiosaka, Osaka, Japan; 7 Division of Molecular Pathology, Center for Neurological Disease and Cancer, Nagoya University Graduate School of Medicine, Nagoya, Japan; University of California, San Diego, United States of America

## Abstract

Amino acid signaling mediated by the activation of mechanistic target of rapamycin complex 1 (mTORC1) is fundamental to cell growth and metabolism. However, how cells negatively regulate amino acid signaling remains largely unknown. Here, we show that interaction between 4F2 heavy chain (4F2hc), a subunit of multiple amino acid transporters, and the multifunctional hub protein girders of actin filaments (Girdin) down-regulates mTORC1 activity. 4F2hc interacts with Girdin in mitogen-activated protein kinase (MAPK)- and amino acid signaling–dependent manners to translocate to the lysosome. The resultant decrease in cell surface 4F2hc leads to lowered cytoplasmic glutamine (Gln) and leucine (Leu) content, which down-regulates amino acid signaling. Consistently, Girdin depletion augments amino acid-induced mTORC1 activation and inhibits amino acid deprivation–induced autophagy. These findings uncovered the mechanism underlying negative regulation of amino acid signaling, which may play a role in tightly regulated cell growth and metabolism.

## Author summary

The mechanistic target of rapamycin complex 1 (mTORC1) protein kinase is a master regulator of cell growth, which senses several extracellular signals, such as growth factors and nutrient levels, to coordinate cell metabolism. The activation of mTORC1 by amino acids requires many proteins such as Rag GTPase, GATOR, and Ragulator. However, how cells negatively regulate amino acid signaling remains largely unknown. In this study, we revealed that an endocytosis-related protein called Girdin negatively regulates amino acid–induced mTORC1 activation via the formation of a complex with 4F2 heavy chain (4F2hc), a subunit of multiple amino acid transporters. We show that Girdin/4F2h complex formation requires growth factor-induced Girdin phosphorylation and amino acid–induced 4F2hc ubiquitination. We also find that the complex promotes the internalization of 4F2hc from the plasma membrane to the lysosomes. The subsequent decrease of 4F2hc in the cell surface results in a lower cytoplasmic glutamine and leucine content, which then down-regulates amino acid–induced mTORC1 activation. These findings uncover the mechanism underlying negative regulation of mTORC1 signaling, which may play a role in tightly regulated cell growth and metabolism.

## Introduction

Cells respond to extracellular stimuli through multiple signaling pathways, which govern and coordinate various cellular activities. Because of their vital roles, these signaling pathways need to be precisely controlled so that cells can respond appropriately to the external cues and maintain cell homeostasis [1]. Cells have several negative regulatory mechanisms to adjust their sensitivity to receptor tyrosine kinase (RTK) and G-protein coupled receptor (GPCR) pathway signals. For example, cells endocytose and subsequently degrade the receptors in the lysosomes in a process called “receptor down-regulation” that makes the cells less responsive to extracellular ligands [2,3].

In recent years, mechanisms by which amino acids function to control several cellular processes have attracted considerable attention [4]. Amino acids activate the mechanistic target of rapamycin complex 1 (mTORC1), which is a signaling complex composed of a conserved serine-threonine kinase mechanistic target of rapamycin (mTOR), Raptor, mammalian lethal with SEC13 protein 8 (mLST8), Deptor, and the proline-rich Akt substrate of 40 kDa (PRAS40) [5]. mTORC1 is essential for multiple biological processes, such as cell growth, anabolism, and autophagy [6]. Notably, mTORC1 signaling is frequently deregulated in several diseases, including cancer and diabetes, which makes it an attractive target for drug discovery [7].

The mTORC1 pathway integrates amino acid and growth factor signaling by distinct mechanisms, in which several small guanosine triphosphatases (GTPases) play important roles. For example, growth factor stimulation activates mTORC1 via the GTPase Rheb, whereas amino acids activate mTORC1 through the heterodimeric Rag, adenosine diphosphate ribosylation factor-1 (Arf1), and Rab1 GTPases [8–11]. Among these, Rag GTPases, which are tightly regulated by the guanine nucleotide exchange factor (GEF) Ragulator and the GTPase-activating protein (GAP) GATOR downstream of amino acid sensors, including Castor, sestrin2, and SAMTOR [12–16], are the most studied. The activated Rag GTPases induce the translocation of mTORC1 to the lysosomes and its activation [9,17]. Although the mechanisms by which cells transmit amino acid signaling to mTORC1 have been elucidated, it remains largely unknown how cells negatively regulate amino acid signaling as observed for the RTK and GPCR signaling pathways. Although two recent studies identified two RagA E3 ligases, RING finger protein 152 and S-phase kinase associated protein 2, which negatively regulate mTORC1 activity through the ubiquitination of RagA, additional negative regulatory mechanisms remain to be discovered [18,19].

We previously identified girders of actin filaments (Girdin) (also known as Gα-interacting vesicle-associated protein) as a substrate for the Akt kinase and an actin-binding protein, which is a multifunctional protein that interacts with several proteins and is involved in cell migration and neuroblast differentiation [20–23]. Recently, we found that Girdin regulates endocytosis via its function as a GAP for dynamin 2 [24]. Intriguingly, Girdin has also been reported to be involved in cell-size control and the regulation of the phosphatidilinositol-3 kinase/Akt pathway [25–27], both of which are closely related to mTORC1 activity, suggesting that it may participate in the mTORC1 signaling pathway.

Here, we describe the identification of 4F2 heavy chain (4F2hc, also known as CD98 heavy chain), a subunit of multiple heterodimeric amino acid transporters, as a Girdin-interacting protein and the role of this protein complex in the negative regulation of mTORC1 activity. Girdin interaction with 4F2hc is regulated by mitogen-activated protein kinase (MAPK) and amino acid signaling, leading to the translocation of 4F2hc to the lysosome and, eventually, decreases in intracellular content of glutamine (Gln) and leucine (Leu) and mTORC1 activity. We propose that this is one of the negative regulatory mechanisms that render cells less responsive to amino acid signaling.

## Results

### Identification of 4F2hc as a Girdin-interacting protein

**Fig 1:**
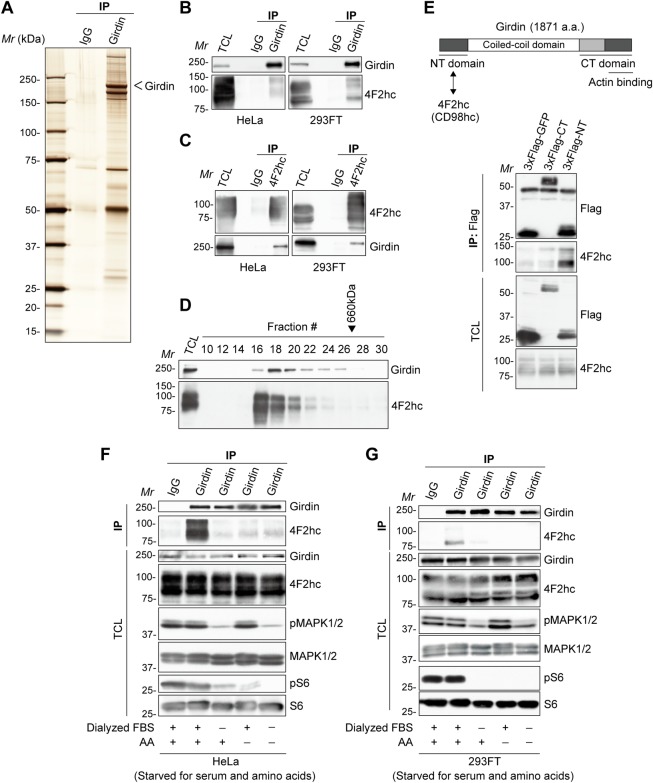
4F2hc interacts with Girdin in amino acid–and serum stimulation–dependent manners. **(A)** Girdin-interacting proteins isolated from mouse brain lysate by co-IP were visualized by silver staining. **(B, C)** HeLa (left panels) and 293FT (right panels) cells were cultured in fresh growth medium with 10% FBS for 30 min before co-IP with indicated antibodies, showing endogenous 4F2hc–Girdin interaction. **(D)** HeLa cell lysates were separated by gel filtration, followed by WB. The elution position of a 660-kDa calibration protein (thyroglobulin) is indicated. **(E)** Primary domain structure of Girdin (upper panel). The Girdin NT domain is responsible for interaction with 4F2hc. 293FT cells were transfected with indicated plasmids, followed by co-IP to determine the domain of Girdin that binds 4F2hc (lower panel). **(F, G)** HeLa (F) and 293FT (G) cells starved for serum and amino acids for 1 h were stimulated with 10% dialyzed FBS, AA, or both for 30 min, followed by co-IP, to detect interaction between 4F2hc and Girdin. AA, amino acids; co-IP, co-immunoprecipitation; CT, carboxyl terminal; FBS, fetal bovine serum; Girdin, girders of actin filaments; IgG, immunoglobulin G; IP, immunoprecipitation; kDa, kilodalton; *Mr*, molecular marker; NT, amino terminal; TCL, total cell lysate; WB, western blot; 4F2hc, 4F2 heavy chain.

To investigate the role of Girdin in mTORC1 signaling, we employed large-scale co-immunoprecipitation (co-IP) to isolate Girdin-interacting proteins in mouse brain lysate. To this end, protein G sepharose beads cross-linked with purified anti-Girdin antibody or normal immunoglobulin G (IgG) were incubated with mouse whole brain lysate. The interacting proteins eluted with acidic buffer (pH 2.8) were visualized by silver staining and further analyzed by a tandem mass spectrometry–based shotgun approach, which identified 4F2hc as one of the Girdin-interacting proteins (Fig 1A, S1 Table). 4F2hc is a type II transmembrane protein that interacts with several amino acid transporters, including LAT1, y^+^LAT1, and xCT, which constitute the heterodimeric amino acid transporter family that is involved in mTORC1 activation [28–32]. Therefore, it was plausible to speculate that the 4F2hc/Girdin interaction participates in amino acid signaling and the regulation of mTORC1 activity.

The interaction of 4F2hc with Girdin as indicated by mass spectrometry was confirmed by reciprocal co-IP in both the HeLa and 293FT cell lines (Fig 1B and 1C). To confirm that Girdin is associated with 4F2hc, protein fractionation by gel filtration was carried out. The elution pattern of Girdin largely overlapped with that of 4F2hc, supporting the hypothesis that Girdin interacts with 4F2hc in vivo (Fig 1D). Mapping of the interacting domains indicated that the Girdin amino terminal (NT) domain, but not the carboxyl terminal (CT) domain, was responsible for the association with 4F2hc (Fig 1E).

Interestingly, 4F2hc/Girdin interaction was not observed in both HeLa and 293FT cells cultured in media depleted of either fetal bovine serum (FBS) or amino acids that are essential for the activation of mTORC1 (Fig 1F and 1G). These data suggested that 4F2hc/Girdin interaction is elaborately regulated through stimulation by multiple nutrients, including growth factors and amino acids.

### MAPK-induced Girdin phosphorylation is required for 4F2hc/Girdin interaction

**Fig 2:**
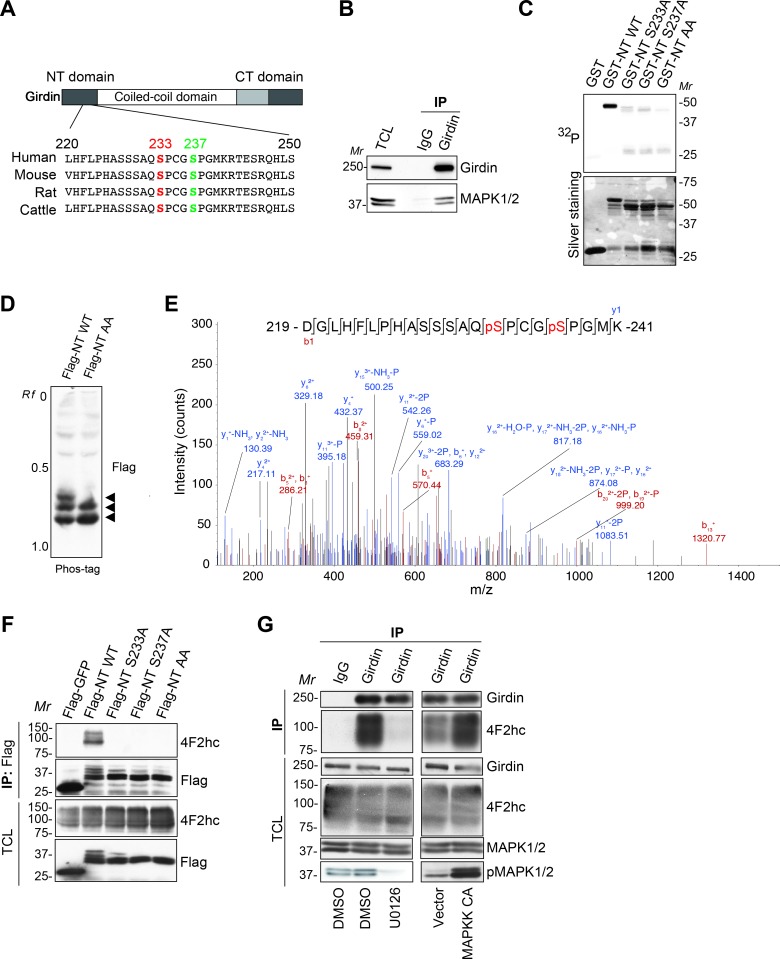
Phosphorylation of Girdin is required for its interaction with 4F2hc. **(A)** Amino acid sequences of Girdins from different species. Conserved serines at positions 233 (red) and 237 (green) that conform to the MAPK substrate motif are highlighted. **(B)** Endogenous interaction between MAPK1/2 and Girdin, as detected by co-IP in 293FT cells. **(C)** In vitro kinase assay showing that MAPK phosphorylates recombinant Girdin NT but not its mutants. **(D)** Lysates from 293FT cells transfected with the indicated plasmids were separated on Phos-tag gel. Arrowheads indicate multiple bands that may represent phosphorylated Girdin NT. **(E)** Lysates from 293FT cells stimulated with FBS in the presence or absence of U0126 were subjected to IP to enrich for endogenous Girdin, followed by the enrichment of phosphopeptides using a Titansphere Phos-TiO kit. The phosphopeptides were analyzed by mass spectrometry. The mass spectrum of a tryptic fragment at m/z 843.3431 (mass error, 0.46 ppm) that matched to the peptide 219-DGLHFLPHASSSAQpSPCGpSPGMK-241 containing phosphorylated S233 and S237 is shown. This phosphopeptide was detected with a high peptide confidence (false discovery rate of less than 1%) in cells stimulated with FBS (Mascot score, 28; expectation value, 0.431) but not in cells pretreated with U0126. **(F)** 293FT cells were transfected with the indicated plasmids, followed by co-IP, showing that mutation of MAPK phosphorylation sites in Girdin disrupts 4F2hc/Girdin interaction. **(G)** 293FT cells starved for serum were pretreated with DMSO or U0126 for 30 min and stimulated with DMEM containing 10% FBS for 30 min, followed by co-IP (left panel). 293FT cells were transfected with indicated plasmids, followed by co-IP to test endogenous 4F2hc/Girdin interaction (right panel). co-IP, co-immunoprecipitation; CT, carboxyl terminal; DMEM, Dulbecco’s Modified Eagle Medium; DMSO, dimethyl sulphoxide; FBS, fetal bovine serum; Flag-NT, Flag-tagged Girdin NT domain; GFP, green fluorescent protein; Girdin, girders of actin filaments; GST, glutathione S-transferase; IgG, immunoglobulin G; MAPK, mitogen-activated protein kinase; MAPKK, MAPK kinase; MAPKK CA, constitutively active mutant of MAPKK; *Mr*, molecular marker; NT, amino terminal; *Rf*, relative mobility; S233, serine at position 233; S237, serine at position 237; WT, wild-type; 4F2hc, 4F2 heavy chain.

Next, we investigated the mechanism underlying the 4F2hc/Girdin interaction downstream of serum and amino acid stimulation. A search through multiple databases, including PhosphoSite (www.phosphosite.org), revealed that serines at positions 233 and 237 (S233 and S237) of Girdin, which are surrounded by consensus MAPK phosphorylation motifs x(S/T)P, are conserved among species and are phosphorylated in cells (Fig 2A). Indeed, endogenous interaction between Girdin and MAPK was shown by co-IP using 293FT cell lysate (Fig 2B). An in vitro kinase assay revealed that the Girdin NT domain, but not its alanine mutants S233A, S237A, and S233A/S237A (further termed “AA”), was phosphorylated by recombinant MAPK, indicating that MAPK directly phosphorylates S233 and S237 of the Girdin NT domain in vitro (Fig 2C). Girdin phosphorylation was also shown by Phos-tag affinity electrophoresis [33], in which Flag-tagged Girdin NT domain (Flag-NT) expressed in 293FT cells migrated as multiple bands, indicating distinct levels of phosphorylation of Flag-NT (Fig 2D). The most slowly migrating band was not observable in the Flag-NT AA mutant, supporting that Girdin is phosphorylated at S233 and S237. To verify that Girdin is phosphorylated by MAPK in vivo, phosphopeptides were enriched from Girdin immunoprecipitates using titanium dioxide (TiO_2_) microparticles, followed by mass-spectrometric analysis. The results demonstrated that Girdin is phosphorylated by MAPK at S233 and S237; a phosphopeptide containing phosphorylated S233 and S237 was detected with high peptide confidence only in cells stimulated with FBS, but not in cells pretreated with the MAPK kinase (MAPKK) inhibitor U0126 (Fig 2E). In addition, co-IP tests showed that all of Flag-NT S233A, S237A, and AA lost the capacity to bind 4F2hc (Fig 2F). Consistently, treatment with U0126 or the expression of a constitutively active mutant of MAPKK (MAPKK CA) inhibited or augmented 4F2hc/Girdin interaction, respectively (Fig 2G). Taken together, these results indicated that MAPK-mediated phosphorylation of Girdin, which occurs downstream of several growth factors, is critical for its interaction with 4F2hc.

### Ubiquitination of 4F2hc by amino acids is essential for its interaction with Girdin

**Fig 3:**
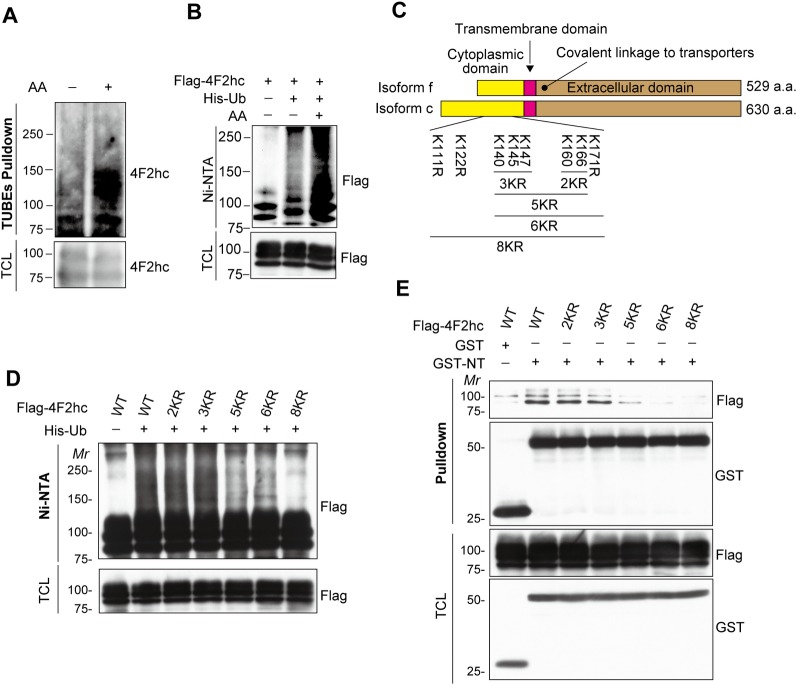
4F2hc/Girdin complex is modulated by ubiquitination of 4F2hc. **(A and B)** 293FT cells transfected with the indicated plasmids were starved for amino acids for 1 h in amino acid–free medium supplemented with dialyzed FBS, followed by stimulation with complete growth medium for 30 min. Ubiquitinated proteins were isolated with glutathione-sepharose beads under a non-denaturing condition **(A)** or Ni-NTA agarose beads under a denaturing condition **(B)** to detect 4F2hc ubiquitination. **(C)** Primary structures of 4F2hc (short and long isoforms) and its mutants used in the study. **(D)** Lysates from 293FT cells transfected with the indicated plasmids were pulled down under denaturing conditions using Ni-NTA agarose beads, showing the ubiquitination of the transfected 4F2hc and its mutants. **(E)** 293FT cells transfected with the indicated combinations of 4F2hc, GST, and GST-NT. The lysates were pulled down with glutathione beads, followed by WB with the indicated antibodies. a.a., amino acids; FBS, fetal bovine serum; Girdin, girders of actin filaments; GST, glutathione S-transferase; His-Ub, histidine-tagged Ub; *Mr*, molecular marker; TUBE, tandem-repeated ubiquitin-binding entity; WB, western blot; WT, wild-type; 4F2hc, 4F2 heavy chain.

A relevant question is how amino acid stimulation modulates the 4F2hc/Girdin interaction. Several reports have indicated that the components of mTORC1 signaling undergo ubiquitination in response to amino acids [18,19]. Therefore, we first tested endogenous 4F2hc ubiquitination by tandem-repeated ubiquitin-binding entities (TUBEs) (also known as ubiquitin traps), which enabled us to measure 4F2hc ubiquitination under a non-denaturing physiological condition (see Materials and methods). We found that 4F2hc was ubiquitinated in 293FT cells, and ubiquitination was significantly increased in response to amino acid stimulation (Fig 3A). Next, we transfected 293FT cells with Flag-tagged 4F2hc and His-tagged ubiquitin and then isolated ubiquitinated proteins using Ni-NTA beads under denaturing conditions. The result showed that amino acid stimulation dramatically promoted 4F2hc ubiquitination (Fig 3B).

We identified eight potential ubiquitination sites in the cytoplasmic domain of 4F2hc from the PhosphoSite database, mutated them in various combinations by substituting lysine (K) residues to arginines (Rs), and compared the ubiquitination of wild-type (WT) 4F2hc and the mutants (Fig 3C and 3D). Simultaneous substitution of five or six potential ubiquitination sites (the corresponding mutant was termed “5KR” or “6KR”) significantly abrogated 4F2hc ubiquitination (Fig 3D). Moreover, the ubiquitination of 4F2hc was almost undetectable in 8KR mutant, which means that all or most of these eight K residues are involved in 4F2hc ubiquitination. Consistently, the 5KR mutant showed a weaker interaction with the Girdin NT domain than its WT counterpart, and the interaction between the Girdin NT domain and 6KR or 8KR mutant was almost undetectable (Fig 3E). These data suggested that the ubiquitination of 4F2hc plays an important role in its interaction with Girdin.

### Girdin negatively regulates amino acid signaling

**Fig 4:**
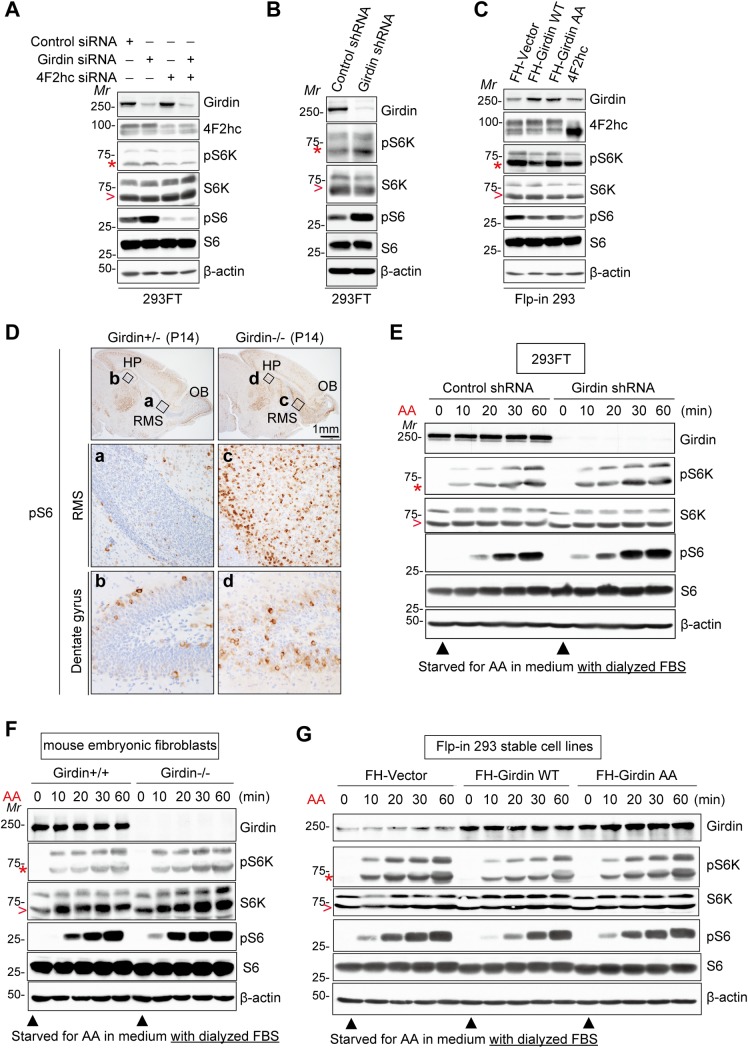
Girdin regulates amino acid stimulation–induced mTORC1 activation through interaction with 4F2hc. **(A and B)** 293FT cells were transfected or transduced with the indicated siRNA or shRNA, respectively, followed by WB to monitor mTORC1 activity. In blot images for S6K1 and pS6K1, the lower bands represent S6K1 or pS6K1, as indicated by arrowhead or asterisk, respectively. Quantitative data are shown in S1A and S1B Fig. **(C)** Basal mTORC1 activity in control Flp-In 293 cells and cells stably overexpressing Girdin WT, Girdin AA, and 4F2hc. Quantitative data are shown in S1C Fig. **(D)** Brain sections from heterogeneous (left) and Girdin knockout (right) P14 mice were stained for pS6. The brown staining indicates pS6 signal. The regions within the black boxes are shown below at a higher magnification. **(E, F, G)** 293FT cells **(E)**, primary mouse embryonic fibroblasts isolated from WT and Girdin-deficient mice **(F)**, or Flp-In 293 cells stably transduced with the indicated constructs **(F)** were starved for amino acids for 1 h in medium supplemented with dialyzed FBS, followed by stimulation with complete medium for the indicated time. mTORC1 activity and Girdin expression were monitored by WB. Quantitative data are shown in S1G–S1I Fig. FBS, fetal bovine serum; FH, Flag-HA epitope; Girdin, girders of actin filaments; HP, hippocampus; *Mr*, molecular marker; mTORC1, mechanistic target of rapamycin complex 1; OB, olfactory bulb; RMS, rostral migratory stream; shRNA, short hairpin RNA; siRNA, small interfering RNA; S6K1, S6 kinase beta1; WB, western blot; WT, wild-type; 4F2hc, 4F2 heavy chain.

Considering that 4F2hc is a component of multiple heterodimeric amino acid transporters [28–30], we next asked whether the 4F2hc/Girdin complex regulates mTORC1 activity in 293FT cells. Consistent with a previous study, small interfering RNA (siRNA)-mediated depletion of 4F2hc inhibited mTORC1 activity, as indicated by the decreased phosphorylation of the ribosomal protein S6 kinase beta1 (S6K1) and S6, which were monitored as readouts for mTORC1 activation (Fig 4A, S1A Fig, S1 Data). In contrast, Girdin depletion by two independent interfering sequences significantly increased the basal level of mTORC1 activity (Fig 4A and 4B, S1A and S1B Fig, S1 Data), which was reciprocally confirmed by the fact that overexpression of WT Girdin, but not its AA mutant that had lost the capacity to bind 4F2hc, significantly suppressed mTORC1 activity (Fig 4C, S1C Fig, S1 Data). Throughout the study, to achieve equal expression levels of exogenously introduced cDNAs and to exclude interclonal differences among cell lines, we utilized the Flp-In system, in which the cDNAs are inserted by homologous recombination into a single genomic recombination target site introduced into the host Flp-In 293 cells (see Materials and methods) to generate cell lines. Additionally, we generated Girdin knockout cells by clustered regularly interspaced short palindromic repeat/CRISPR-associated 9 (CRISPR/Cas9)-mediated genome editing (S1D–S1F Fig). In all of HeLa, 293FT, and Flp-In 293 cells, Girdin knockout cells showed higher basal mTORC1 activity than WT cells. In addition, re-expression of WT Girdin, but not Girdin AA mutant, significantly decreased mTORC1 activity in Girdin knockout Flp-In 293 cells (S1F Fig). Unexpectedly, the overexpression of 4F2hc also inhibited basal mTORC1 activation, which might be due to mislocalization of amino acid transporters or disruption of endogenous amino acid transporter complexes by overexpressed 4F2hc (Fig 4C). In addition, 4F2hc knockdown abrogated Girdin depletion-induced mTORC1 activation, indicating that Girdin regulates mTORC1 activity through 4F2hc (Fig 4A).

The significance of Girdin in mTORC1 regulation was further supported by the observation that neuroblasts in the rostral migratory stream (RMS) and the dentate gyrus (DG) of the brain, the development of which have been shown to be specifically regulated by Girdin [23,34], exhibited significantly high S6 activity in Girdin-deficient as compared to control mice (Fig 4D). This result supported the notion that Girdin suppresses mTORC1 activity.

Next, we examined the kinetics of mTORC1 activation in amino acid–stimulated cells. Girdin depletion significantly augmented mTORC1 activity over time upon amino acid stimulation in 293FT cells cultured in the presence of dialyzed serum (Fig 4E, S1G Fig, S1 Data). This was confirmed in primary mouse embryonic fibroblasts isolated from control and Girdin-deficient mice (Fig 4F, S1H Fig, S1 Data). Overexpression of WT Girdin, but not its AA mutant, significantly inhibited amino acid–induced mTORC1 activation, supporting that Girdin is a negative regulator of mTORC1 in the presence of serum and amino acid signals (Fig 4G, S1I Fig, S1 Data).

This notion was corroborated by the fact that Girdin depletion inhibited autophagy induced by amino acid withdrawal, as shown by the reduced lipidation of microtubule-associated protein light chain 3 (LC3) (S2A Fig, S1 Data). In this experiment, we also tested the effect of the lysosome inhibitor Bafilomycin A1, which inhibits the late phase of autophagy, including autophagosome–lysosome fusion and autolysosome acidification [35]. In cells treated with Bafilomycin A1, Girdin knockdown still inhibited amino acid starvation–induced autophagy, indicating that Girdin affects the early autophagy phase, as mTORC1 reportedly does (S2A Fig) [36]. Furthermore, the overexpression of WT Girdin, but not its AA mutant, accelerated autophagy, as indicated by the decreased appearance of green fluorescent protein (GFP)-LC3 puncta (S2B–S2D Fig, S1 Data). These findings suggested that Girdin does not directly regulate S6K1 and S6 activities but rather affects the entire mTORC1 pathway. Collectively, the data indicated that the 4F2hc/Girdin complex is involved in the negative regulation of amino acid and mTORC1 signaling pathways.

### Girdin induces internalization of 4F2hc by lysosomes

**Fig 5:**
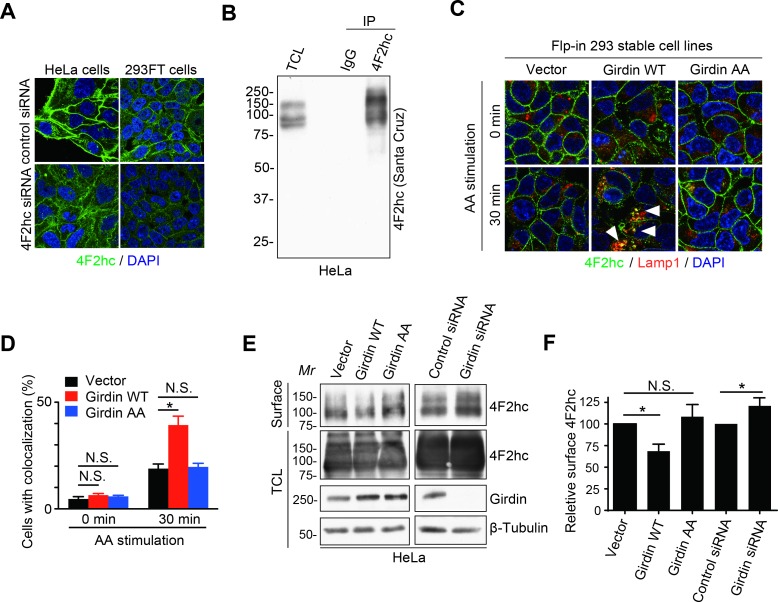
Girdin promotes 4F2hc translocation to the lysosomes. **(A)** In-house-developed 4F2hc antibody was used for IF in HeLa cells and 293FT cells; staining intensity was markedly reduced in 4F2hc knockdown cells. **(B)** In-house-developed 4F2hc antibody was used for IP in HeLa cells, and the sample was detected by commercial 4F2hc antibody to verify the quality of the antibody. **(C)** Flp-In 293 cells stably transduced with the indicated plasmids were starved for amino acids for 1 h and stimulated with amino acids for 30 min, followed by immunofluorescence staining for 4F2hc (green) and Lamp1 (red). Arrowheads indicate the localization of 4F2hc in the lysosome. **(D)** Quantification of the cells shown in **(C)** with 4F2hc localized on the lysosome (100 cells from three independent experiments). The data are presented as means ± SEs. *P < 0.05. The data underlying this figure can be found in S1 Data. **(E)** Cell surface proteins of HeLa cells transfected with the indicated siRNAs or plasmids were biotinylated and isolated, followed by WB with the indicated antibodies. **(F)** Quantification of the bands intensity of cell surface 4F2hc from 3 independent experiments is shown. The data are presented as means ± SEs. *P < 0.05. The value of the surface 4F2hc in the cells transfected with empty vector or control siRNA were set as 100. The data underlying this figure can be found in S1 Data. Girdin, girders of actin filaments; IgG, immunoglobulin G; *Mr*, molecular marker; N.S., not significant; siRNA; small interfering RNA; WB, western blot; WT, wild-type; 4F2hc, 4F2 heavy chain.

Given that Girdin was previously reported as a critical regulator of intracellular membrane trafficking [24], we hypothesized that Girdin-mediated negative regulation of mTORC1 activity was attributed to 4F2hc internalization. Immunofluorescence analysis, in which the specificity of the 4F2hc antibody used (clone HBJ 127) [37] was verified by the knockdown of endogenous 4F2hc (Fig 5A) and an IP test followed by western blot (WB) analysis using a different, commercially available anti-4F2hc antibody (Fig 5B), showed that 4F2hc localized to the plasma membrane. The expression of WT Girdin, but not the AA mutant, promoted 4F2hc translocation to the lysosomes after amino acid stimulation (Fig 5C and 5D, S1 Data). Consistently, cell fractionation showed that Girdin overexpression and depletion decreased and increased the cell surface level of 4F2hc, respectively (Fig 5E and 5F, S1 Data). Together, these results suggested that the 4F2hc/Girdin interaction promotes the translocation of 4F2hc from the plasma membrane to the lysosome.

### 4F2hc/Girdin complex decreases intracellular Gln and Leu contents

**Fig 6:**
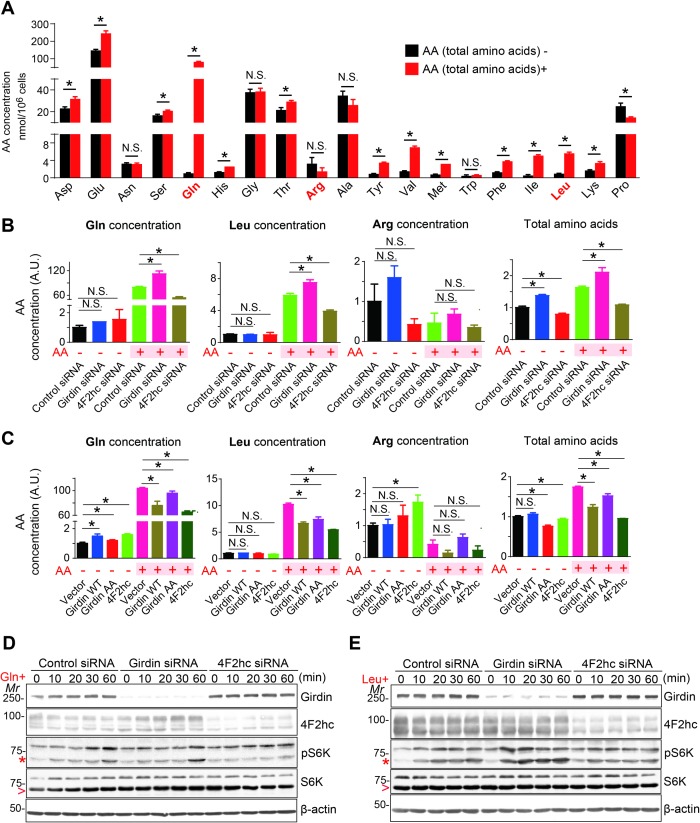
Girdin negatively regulates amino acid signaling through decreasing intracellular Gln and Leu contents. **(A)** 293FT cells starved for amino acids for 1 h were stimulated with total amino acids (AA+) for 10 min. Amino acid concentrations were measured with an HPLC system. Quantification of each amino acid concentration from 3 independent experiments is shown; the data are presented as means ± SEs. *P < 0.05. The data underlying this figure can be found in S1 Data. **(B, C)** 293FT cells transfected with indicated siRNA **(B)** or Flp-in 293 cells stably expressing empty vector, Girdin WT, Girdin AA, and 4F2hc **(C)** were starved for amino acids for 1 h and stimulated with total amino acids for 10 min. Quantification of each amino acid concentration from 3 independent experiments is shown; the data are presented as means ± SEs. *P < 0.05. The values in control cells (control siRNA or empty vector) starved for amino acids were set as 1. The data underlying this figure can be found in S1 Data. **(D, E)** 293FT cells transduced with control or Girdin shRNA were starved for 1 h in amino acid–free medium and stimulated with Gln **(D)** or Leu **(E)** for the indicated times, followed by WB. Note that the activation of mTORC1 was observed at 60 min in Gln stimulated cells **(D)**. AA, amino acids; Arg, arginine; A.U., arbitrary unit; Gln, glutamine; Leu, leucine; Girdin, girders of actin filaments; *Mr*, molecular marker; mTORC1, mechanistic target of rapamycin complex 1; N.S., not significant; shRNA, short hairpin RNA; siRNA, small interfering RNA; WB, western blot; WT, wild-type; 4F2hc, 4F2 heavy chain.

Considering that Girdin down-regulated the cell surface level of 4F2hc via internalization and previous reports showing that intracellular amino acids are crucial for mTORC1 activation [9,14–16], we next asked whether the 4F2hc/Girdin complex regulates mTORC1 activity through the modulation of intracellular amino acid contents. Previous studies have shown that Gln, Leu, and arginine (Arg) are three intracellular amino acids that activate mTORC1 [10,38,39]. Our comprehensive measurement of cytosolic amino acids showed that 10-min stimulation with total amino acids, which is sufficient to activate mTORC1, led to increases in Gln and Leu (Fig 6A, S3A and S3B Fig). Interestingly, while most amino acids increased in concentration upon total amino acid stimulation, Arg did not change (Fig 6A, S1 Data), which may be due to the activity of some amino acid exchangers that mediate Arg efflux [40]. These results confirmed the correlation between mTORC1 activation and intracellular amino acid contents—at least those of Gln and Leu—in our experimental setup.

Next, we examined the effects of Girdin and 4F2hc depletion and overexpression on intracellular amino acid contents (Fig 6B and 6C, S3A and S3B Fig, S1 Data). In cells stimulated with total amino acids, Girdin knockdown led to significantly increased Gln and Leu contents, in contrast to 4F2hc depletion, which led to decreases in the two amino acids (Fig 6B, S3A Fig, S1 Data). Neither Girdin nor 4F2hc knockdown had an effect on intracellular Arg under the same condition. These results were consistent with our finding that Girdin knockdown increased amino acid–stimulated mTORC1 activation, whereas 4F2hc knockdown decreased it (Fig 4A), suggesting that Girdin and 4F2hc modulate mTORC1 activity through the regulation of intracellular Gln and Leu contents. Overexpression of Girdin and 4F2hc led to significant decreases in Gln and Leu, but not Arg, in cells stimulated with total amino acids (Fig 6C, S3B Fig, S1 Data). Overexpression of Girdin AA mutant also resulted in decreased Gln and Leu contents, but less significantly than WT. These results are consistent with our findings that overexpression of Girdin WT and 4F2hc inhibited mTORC1 activation. In addition, because 4F2hc guides several amino acid transporters to localize on the cell membrane to regulate many amino acids’ uptake, we also found that the knockdown of 4F2hc significantly decreases total amino acid contents in cells (Fig 6B, S1 Data), which further verified that our test is reliable.

**Fig 7:**
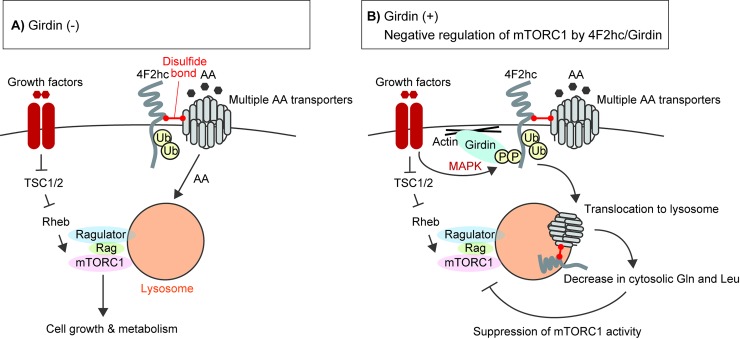
Schematic model of the negative regulation of amino acid signaling by the 4F2hc/Girdin complex. **(A)** In the absence of the 4F2hc/Girdin complex, various growth factor and amino acid signaling pathways converge on mTORC1 to regulate its activity and cell metabolism. **(B)** In the presence of Girdin, it interacts with 4F2hc depending on MAPK activity and amino acid stimulation, which promotes endocytosis of 4F2hc into the lysosome, leading to changes in intracellular Gln, Leu, and mTORC1 activity. AA, amino acids; Girdin, girders of actin filaments; Gln, glutamine; Leu, leucine; MAPK, mitogen-activated protein kinase; mTORC1, mechanistic target of rapamycin complex 1; TSC, tuberous sclerosis; Ub, ubiquitin; 4F2hc, 4F2 heavy chain.

In these experiments, Gln and Leu were very low in starved cells (Fig 6A, S3 Fig), which indicated the significance of uptake of these amino acids across the plasma membrane in determining cellular mTORC1 activation (Fig 6B and 6C). In addition, although there were some differences in Gln and Arg concentration in starved Girdin or 4F2hc-overexpressing cells, the concentration was also very low (Fig 6C, S3 Fig). Supporting this notion, we found that Girdin and 4F2hc depletion significantly up-regulated and inhibited mTORC1 activation, respectively, in cells stimulated with either Gln or Leu (Fig 6D and 6E). Notably, the kinetics of mTORC1 activation induced by Gln and Leu stimulation were different: Leu stimulation activated mTORC1 quickly, whereas Gln-stimulated activation of mTORC1 required about 1 h stimulation, as previously reported [10]. Altogether, the data implied that Girdin down-regulates the cell surface level of 4F2hc via endocytosis, which subsequently decreases intracellular Gln and Leu contents to negatively regulate mTORC1 activation (Fig 7).

## Discussion

Our present study identified a novel negative regulatory mechanism for amino acid signaling. This process is mediated by Girdin–4F2hc interaction, which is regulated by Girdin phosphorylation and 4F2hc ubiquitination downstream of MAPK and amino acid stimulation, respectively. The interaction between Girdin and 4F2hc modulates the cell surface level of 4F2hc through the regulation of 4F2hc endocytosis, which subsequently changes intracellular Gln and Leu contents to negatively regulate amino acid signaling. The involvement of Arg in Girdin-mediated mTORC1 regulation remains unclear. In addition, because the changes of mTORC1 activation by Girdin knockdown were relatively modest (Fig 4E–4G), additional mechanisms apart from Girdin–4F2hc interaction could also play a role in mTORC1 regulation.

An interesting finding from the present study is that the formation of the 4F2hc/Girdin complex required signals from both growth factors and amino acids, both of which are known to be essential for mTORC1 activation. This finding suggested that the 4F2hc/Girdin complex integrates signals from various factors, such as nutrients, to counteract the anabolic and biosynthetic pathways induced by these factors. In accordance herewith, 4F2hc is a hub protein that interacts with not only transporters for essential amino acids such as LAT1 and y^+^LAT1 but also a cystine transporter, xCT, and a glucose transporter, GLUT1 [32,41]. This led us to speculate that the 4F2hc/Girdin complex may also be involved in the control of cystine-mediated intracellular redox balance or glucose metabolism.

We showed that MAPK-mediated Girdin phosphorylation is crucial for the endocytosis and lysosomal localization of 4F2hc. This finding is in accordance with a previous report that Girdin functions to activate dynamin GTPase, which is essential for the pinching off of clathrin-coated vesicles and their endocytosis [24]. However, the upstream mechanism(s) of amino acid–induced 4F2hc ubiquitination, which also has an effect on the formation of the 4F2hc/Girdin complex (Fig 3), remains unknown. One obvious candidate for the ubiquitination of 4F2hc is the membrane-associated RING-CH E3 ubiquitin ligase 1 and 8 (MARCH1/8), which was previously shown to ubiquitinate 4F2hc [42]. Consistent with our finding, one study showed that MARCH1/8-mediated ubiquitination of 4F2hc leads to its translocation from the plasma membrane to the lysosome [43]. In addition, another study reported that 4F2hc translocates to the lysosome through binding to the lysosomal protein, LAPTM4b [44]. We speculate that Girdin promotes amino acid–stimulated 4F2hc endocytosis, and the internalized 4F2hc may translocate to the lysosome via interaction with LAPTM4b.

To clarify the mechanism of Girdin–4F2hc interaction in regulation of mTORC1 activity, we investigated the role of Girdin and 4F2hc on intracellular amino acids. Although some studies have reported an essential role of lysosomal amino acids in mTORC1 activation [45], a very recent study showed that 1-h amino acid starvation led to a significant decrease in essential amino acids in whole cell lysate but not the lysosome fraction, which implicated that lysosomal amino acids may not be a major driving force for mTORC1 activity [46]. This was consistent with the fact that most amino acid sensors identified so far, such as Castor, sestrin2, and SAMTOR, are located in the cytosol [14–16]. Therefore, we focused on the effects of Girdin and 4F2hc on cytosolic amino acids, specifically, Leu, Gln, and Arg, all of which regulate mTORC1 activity. Girdin depletion–mediated mTORC1 activation was accompanied by an increase in intracellular Gln and Leu contents, whereas no obvious changes in Arg were detected among control and Girdin or 4F2hc knockdown cells under the given culture conditions. Based on these findings, we speculated that Girdin negatively regulates amino acid signaling via modulating 4F2hc endocytosis and the cytosolic contents of Gln and Leu.

The significance of the negative regulation of amino acid signaling by 4F2hc/Girdin complex in vivo has not been revealed in this study. In embryonic development and homeostasis in adult tissues, mTORC1 activity needs to be tightly controlled; deregulation of mTORC1 leads to stem cell aging, reduced tissue regeneration capacity, and the progression of diseases such as cancer [47,48]. Further studies on animal models should be devoted to clarifying the biological context in which the 4F2hc/Girdin complex functions. One potential role of Girdin-mediated mTORC1 regulation was suggested by our finding that mTORC1 activity is significantly up-regulated in neuroblasts in the RMS and DG in the brain of Girdin-deficient mice (Fig 4D). We and others have previously reported that Girdin is essential for the development of postnatal brain and adult neurogenesis, in which it controls the differentiation and migration of newly generated neuroblasts in the RMS and DG [23,34]. We therefore speculate that the negative regulatory effect of Girdin on mTORC1 may contribute to the proper development of those brain regions in the developmental and adult stages. Girdin is expressed in some types of human malignancies, in which it may have a role in maintaining appropriate activation of mTORC1. Further experiments are required to verify the physiological functions of Girdin-mediated mTORC1 regulation.

## Materials and methods

### Ethics statement

All animal protocols were approved by the Animal Care and Use Committee of Nagoya University Graduate School of Medicine (Approval number 29312). All in vivo experiments were performed in compliance with Nagoya University’s Animal Facility regulations.

### Cell culture, transfection, and RNA interference

The HeLa cell line was purchased from the American Tissue Type Culture (Rockville, MD). 293FT and Flp-in 293 cells were purchased from Invitrogen (Carlsbad, CA). Primary mouse embryonic fibroblasts were isolated from WT and Girdin-deficient mouse E13.5 embryos by chemical digestion followed by mechanical disaggregation. All of the cells were cultured in Dulbecco’s Modified Eagle Medium (DMEM, Nacalai Tesque, Kyoto, Japan) supplemented with 10% FBS. Amino acid–free DMEM (Cell Science and Technology Institute, Inc., Sendai, Japan) supplemented with 10% dialyzed FBS (Thermo Fisher Scientific) was used to starve cells. siRNAs or plasmids were transfected into cells using Lipofectamine 2000 (Invitrogen) according to the manufacturer’s instructions. PolyFect (Qiagen, Hilden, Germany) was used to obtain high-level expression in HeLa cells. The target sequences of the siRNAs, the specificities of which have been previously demonstrated [20], were as follows: Girdin, 5′-AAGAAGGCTTAGGCAGGAATT-3′; 4F2hc, 5′-CAGATCCTGAGCCTACTCGAA-3′. For short hairpin RNA (shRNA)-mediated knockdown of Girdin, Girdin target sequence (5′-GGAACAAACAAGATTAGAA-3′) was inserted into the pSIREN-RetroQ retroviral shRNA expression vector (Clontech Laboratories, Palo Alto, CA). A control shRNA vector provided by Clontech Laboratories was used as a negative control. The production of retroviral supernatants by GP2-293 packaging cells (Clontech Laboratories) is described below.

### Antibodies and reagents

The following antibodies were used in this study: anti-Girdin (R&D Systems, Minneapolis, MN, #AF5345, for WB and IP), anti-4F2hc (H300, Santa Cruz Biotechnology, Santa Cruz, CA, for WB, clone MEM-108, Biolegend [#315602] for IP, and mouse monoclonal anti-4F2hc [clone HBJ 127] for IF), anti-glutathione S-transferase (GST) (Santa Cruz Biotechnology, #sc-459), anti-Myc (clone 9E10, Santa Cruz Biotechnology), anti-poly-histidine (clone HIS-1, Sigma, St. Louis, MO), anti-Flag (clone M2, Sigma), anti-β-actin (clone AC-74, Sigma), normal mouse IgG (Millipore, Milford, MA, #12–371), and normal sheep IgG (Millipore, #12–515). Antibodies to pS6K (Thr389) (#9205), S6K (#9202), S6 (#2217), pS6 (Ser240/244) (#2215), MAPK (#9102), pMAPK (Thr202/Tyr204) (#9106), Lamp1 (#9109), mTOR (#2983), and LC3B (#2775) were purchased from Cell Signaling Technology (Danvers, MA). Flag M2 affinity gel, adenosine 5′-triphosphate (ATP), and amino acids were purchased from Sigma. Phos-tag Acrylamide (Wako, Saitama, Japan) was used for the generation of the Phos-tag gel to analyze protein phosphorylation in cells.

### Generation of Girdin knockout cells using CRISPR/Cas9 genome editing

The 20 nucleotide guide sequences targeting human Girdin were designed using the CRISPR design tool at http://www.genome-engineering.org/crispr/ and cloned into a bicistronic expression vector pX459 (Addgene #48139). The guide sequence targeting exon 9 of human Girdin was 5′-GGAAGTGACTGATATGTCGC-3′. The single guide RNAs (sgRNAs) in the pX459 vector (1 μg) were transfected into targeting cells in a 3.5-cm dish using Lipofectamine 2000 (Invitrogen) according to the manufacturer’s instructions. Twenty-four hours post-transfection, the cells were trypsinized and seeded in a 10-cm dish; puromycin (2 μg/mL) was added 24 h later for selection. Forty-eight hours after selection, the cells were trypsinized and seeded into a 96-well plate (4 cells per well). Single clones were expanded and screened for Girdin expression by protein immunoblotting.

### Purification and identification of Girdin-interacting proteins

We isolated Girdin immunocomplex by co-IP. One hundred micrograms of anti-Girdin antibody or normal IgG was cross-linked onto 50 μL of protein G beads using disuccinimidyl suberate (DSS, Thermo Scientific Pierce #21658) according to the manufacturer’s instructions. To prepare mouse brain lysate, adult mouse brains without cerebellum were lysed in IP lysis buffer (20 mM Tris-HCl, 120 mM NaCl, 0.8% Triton X-100, 1 mM EDTA, pH 7.4) using Dounce homogenizer (1 mL of lysis buffer was used to lyse 1 mouse brain). The brain lysate was cleared by centrifugation at 100,000 *g* for 1 h at 4°C, and the supernatant was collected as whole mouse brain lysate. The lysate was precleared by incubation with Protein G beads for 1 h. One hundred milligrams of precleared brain lysate was incubated with 50 μL of Protein G beads cross-linked with the indicated antibody. After overnight rotation at 4°C, the beads were extensively washed with 10 mL of lysis buffer and 10 mL of PBS, followed by elution of the protein complex with 190 μL of acidic buffer (Thermo Scientific Pierce #210004) for 5 min at room temperature. The eluate was neutralized by adding 10 μL of 1M Tris-HCl (pH 9.5). Twenty-five microliters of eluate were separated by sodium dodecyl-polyacrylamide gel electrophoresis (SDS-PAGE) and detected by silver staining (SilverQuest Silver Staining Kit, Invitrogen) following the manufacturer’s instructions. For the identification of proteins included in the eluate, the whole eluates were digested with Trypsin Gold (Promega, Madison, WI) for 16 h at 37°C after reduction, alkylation, demineralization, and concentration, followed by analysis on the Orbitrap Fusion mass spectrometer (Thermo Fisher Scientific, San Jose, CA).

### Measurement of intracellular amino acids

All procedures were performed on ice or at 4°C with prechilled buffers. Samples for amino acid concentration measurement were prepared as previously described, with minor modifications [49]. Briefly, cells cultured to 90%–100% confluence in a 6-cm dish were quickly washed twice with PBS and scraped into 1 mL of 80% methanol in water. After centrifugation at 15,000 rpm for 15 min, the supernatants were collected, and amino acids were measured using an Agilent 1100 HPLC System (Wako, Osaka, Japan). For each sample, replicate sets of cells that were prepared and treated identically were used for amino acid measurement and cell counting, respectively.

### Plasmids

cDNA encoding human 4F2hc28 was inserted into the pcDNA3.1 (Invitrogen), pcDNA3-Flag-HA (1436, Addgene, Cambridge, MA), or pcDNA5/FRT (Invitrogen) vector. A cDNA fragment encoding the 4F2hc cytoplasmic domain (102–184) was inserted into the pGEX-5X-2 vector (GE Healthcare, Waukesha, WI). The construction of plasmids pGEX-5X-2-GST-Girdin-NT (1–256), pET-21a-Girdin-NT(1–256)-His6, pEF-BOS-GST, and pEF-BOS-GST-Girdin-NT was previously described [18]. cDNA encoding full-length human Girdin was inserted into pcDNA5/FRT-Flag-HA and pBICEP-CMV2 vectors (Sigma). GFP and Girdin-NT cDNAs were inserted into the pRetroQ-3xFlag vector. Ubiquitin cDNA (Myc-Ub), MAPKK DA, and GFP-LC3 were gifts from Keiji Tanaka (Tokyo Metropolitan Institute of Medical Science), Yukiko Gotoh (The University of Tokyo), and Toyoshi Fujimoto (Nagoya University), respectively. Histidine-tagged Ub (His-Ub) was kindly provided by Dr. Hui-Kuan Lin (University of Texas M.D. Anderson Cancer Center).

### Retrovirus infection

To generate stable Girdin knockdown 293FT cells, 24 μg of either control or Girdin shRNA and 4 μg of vesicular stomatitis virus G protein (pVSV-G) vector (Clontech) were cotransfected into GP2-293 packaging cells (Clontech Laboratories). Twenty-four hours after the transfection, the medium was replaced with 5 mL of fresh DMEM containing 10% FBS and the cells were further cultured at 32°C to facilitate virus production. HeLa cells were infected with virus-containing supernatants harvested 48 h post-transfection, followed by selection with 2 μg/mL puromycin.

### Immunoprecipitation and WB analysis

Cells were lysed in IP lysis buffer (20 mM Tris-HCl, 120 mM NaCl, 0.8% Triton X100, 1 mM EDTA, pH 7.4) supplemented with Complete Mini protease inhibitor and PhosSTOP phosphatase inhibitor cocktails (Roche). The lysates were cleared via centrifugation at 12,000 × *g* for 10 min and the supernatants were incubated with 2 μg of the appropriate primary antibodies or normal IgG on a rotator at 4°C overnight, followed by the addition of 20 μL of protein A or G Sepharose beads (Sigma) at 4°C for 3 h. Then, the beads were washed three times with IP lysis buffer, and the protein complex was eluted using 100 μL of 1× SDS sample buffer. For the detection of endogenous 4F2hc/Girdin interaction, the medium was replaced with fresh DMEM containing 10% FBS for 0.5–1 h before preparation of cell lysates. For IP with anti-Flag antibody, the cell lysates were incubated with 20 μL of ANTI-FLAG M2 Affinity Gel (Sigma) for 3 h. Then, the beads were washed three times with IP lysis buffer followed by elution of the protein complex using 100 μL of 1× SDS sample buffer.

For WB analysis, samples separated by SDS-PAGE were transferred onto polyvinylidene difluoride membranes. The membranes were blocked with 5% skimmed milk in TBS-T (Tris-buffered saline containing 0.1% Tween-20) and incubated with primary antibodies, followed by detection by using horseradish peroxidase-conjugated antibodies (Dako, Carpinteria, CA). Band intensities were quantified using ImageJ (NIH, Bethesda, ML).

### Identification of endogenous Girdin phosphorylation by mass spectrometry

To identify endogenous Girdin phosphorylation, 293FT cells treated with or without U0126 for 30 min were stimulated with DMEM containing 10% FBS for 20 min, followed by IP to enrich endogenous Girdin. The IP products, including the beads, were denatured with 200 μL of 7 M guanidine hydrochloride. After centrifugation for 5 min at 10,600 × *g* at room temperature, the supernatant was transferred to a new 1.5-mL tube and digested with trypsin for 16 h at 37°C after reduction (4 mM DTT), alkylation (8 mM iodoacetamide), demineralization, and concentration (methanol/chloroform). The phosphopeptides were concentrated using a Titansphere Phos-TiO kit (GL Sciences) according to the manufacturer’s instructions, followed by analysis on an Orbitrap Fusion mass spectrometer (Thermo Fisher Scientific, San Jose, CA).

### Protein expression and purification

For the purification of GST fusion proteins, BL21 competent cells (Stratagene, Santa Clara, CA) transformed with the indicated plasmids were cultured in 2× YT medium containing ampicillin (100 μg/mL) at 37°C until the absorbance at 600 nm (A600) reached 0.6–0.8. Protein expression was induced by adding 100 μM isopropyl beta-d-thiogalactoside (IPTG), followed by continuous culture at 25°C for an additional 4 h. The cell pellets were suspended in homogenizing buffer (20 mM Tris-HCl, pH 8.0, 1 mM EDTA, 1 mM DTT, 10% sucrose) supplemented with complete protease inhibitor cocktail (Roche) and sonicated extensively. The lysates were cleared via centrifugation at 37,000 rpm for 1 h and applied to a column of Glutathione Sepharose 4B beads with a 1-mL bed volume (GE Healthcare) equilibrated with 20 mL of TED buffer (20 mM Tris-HCl, 1 mM EDTA, 1 mM DTT, pH 8.0). The column was washed extensively with 10 mL of TED buffer and the GST fusion protein was eluted using elution buffer (10 mM glutathione in TED buffer), followed by dialysis with TED buffer.

### In vitro kinase assay

The phosphorylation assay was performed as previously described [14]. In brief, MAPK was reacted with the recombinant proteins of Girdin-NT WT and mutants (S233A, S237A, and AA) in 50 μL of reaction mixture (20 mM MOPS, pH 7.2, 25 mM β-glycerol phosphate, 5 mM EGTA, 1 mM sodium orthovanadate, 1 mM DTT, 13.5 mM MgCl2, 90 μM ATP containing [g-32P]ATP [Perkin Elmer, NEG502Z], 1 ng/μl active mouse MAPK [Erk2, Millipore], and 0.8 μM purified Girdin-NT) for 10 min at 32°C. Then, the reaction mixtures were boiled in SDS sample buffer and subjected to SDS-PAGE and silver staining. The radiolabeled proteins were visualized with an image analyzer (Typhoon FLA 9000; GE Healthcare Life Sciences).

### Ubiquitination assay

Two methods were used to test in vivo ubiquitination of 4F2hc. For the detection of endogenous 4F2hc ubiquitination, we exploited the recently developed TUBEs approach to capture ubiquitinated proteins [50,51]. 293FT cells treated with MG132 (10 μM, Sigma) for 4 h were starved for amino acids for 1 h and stimulated with total amino acids for 1 h. The cells were then lysed with 500 μL of IP lysis buffer supplemented with Complete Mini protease inhibitor and PhosSTOP phosphatase inhibitor cocktails (Roche) and 25 μg of GST-TUBE2 (LifeSensors, #UM102). Cleared cell lysates were incubated with 50 μL of Glutathione Sepharose 4B beads for 2 h. The beads were washed three times with IP lysis buffer, followed by elution of the protein complex by 100 μL 1× SDS sample buffer and WB analysis using anti-4F2hc antibody.

For the detection of exogenous 4F2hc ubiquitination, 293FT cells transfected with 4F2hc (1.5 μg) and His-Ub (3 μg) plasmids were treated with MG132 (10 μM) for 4 h. Then, the cells were washed twice with ice-cold PBS, scraped off the plates in the PBS, and collected by centrifugation at 600 × *g* for 5 min. The cell pellets were lysed in buffer C (6 M guanidine-HCl, 0.1 M Na_2_HPO_4_/NaH_2_PO_4_, 10 mM imidazole, pH 8.0) and sonicated. The whole cell extracts were mixed with 100 μL of Ni-NTA agarose beads (Qiagen) at 4°C overnight. The Ni-NTA beads were washed twice with buffer C, once with buffer D (1:3 mixture of buffer C: buffer E), and once with buffer E (25 mM Tris-HCl, 20 mM imidazole, pH 6.8). The bound proteins were eluted by boiling in 1× SDS loading buffer containing 300 mM imidazole and resolved by SDS-PAGE, followed by WB analysis.

### Isolation of cell surface protein

Cells were quickly washed twice using ice-cold PBS on ice and then incubated with 250 μg/mL of sulfosuccinimidyl-2-(biotinamido)ethyl-1,3-dithiopropionate (sulfo-NHS-SS-Biotin; Thermo Scientific) at 4°C for 30 min. The cells were washed three times with ice-cold PBS and lysed in IP lysis buffer. The cell lysates were incubated with 20 μL of NeutrAvidin agarose (Thermo Scientific) for 1 h, followed by three washes with IP lysis buffer. Bound proteins were eluted by boiling in 1× SDS loading buffer and were detected by WB analysis.

### Immunofluorescence staining

Immunofluorescence studies were performed as previously described [18]. Cells were plated on glass base dishes (Iwaki, Osaka, Japan), fixed, and stained with the indicated antibodies. The cells were imaged using a confocal laser-scanning microscope (LSM700, Carl Zeiss, Oberkochen, Germany).

### Immunohistochemistry

Immunohistochemistry was performed as previously described [23]. Mouse brains were perfused with 4% paraformaldehyde in 0.1 M phosphate buffer, postfixed in the same fixative overnight, and cut into 50–60-μm sections on a microslicer (VT1200S, Leica, Heidelberg, Germany). Tissue sections were deparaffinized and rehydrated, and antigens were retrieved by boiling in target retrieval solution pH 9 (Dako) for 30 min. After washing with PBS containing 0.05% Tween-20, Protein Blocking Agent (Dako) was used to cover slides for 30 min, and then anti-pS6 antibody (Cell Signaling Technology) diluted (1:100) in 0.1% BSA/PBS buffer was applied to the slides overnight at 4°C. After washing, sections were treated with 3% hydrogen peroxide/ethanol solution for 15 min at room temperature and incubated with horseradish peroxidase-labeled anti-rabbit IgG secondary antibodies (EnVision System, Dako) for 30 min at room temperature, followed by signal detection with diaminobenzidine solution.

### Data analysis

The data are presented as means ± standard errors (SEs). Statistical significance was evaluated using Student *t* test. *P* < 0.05 was regarded as significant. All experiments were repeated at least 3 times.

## Supporting information

S1 Table Girdin-interacting proteins identified by IP and mass spectrometry. Girdin, girders of actin filaments.
(XLSX)

S1 Data Raw data used for quantification in this work. (XLSX)

S1 Fig Girdin negatively regulates basal mTORC1 activity. **(A–C)** Band intensities for pS6K1 and S6K1, and pS6 and S6 in Fig 4A–4C were quantified, and the ratios of pS6K1 to S6K1 and pS6 to S6 are presented as the mean ± SE in **(A)** (related to Fig 4A), **(B)** (related to Fig 4B), and **(C)** (related to Fig 4C). Values in control cells were set as 1. All experiments were repeated 3 times. The data underlying this figure can be found in S1 Data. **(D, E)** Girdin knockout cells were generated by using the CRISPR/Cas9 system. Lysates from the WT parent cells and Girdin knockout cells were analysed by WB to detect the basal activation level of mTORC1. **(F)** Girdin WT or AA mutant was re-expressed in Girdin knockout Flp-In 293 cells, followed by detection of basal mTORC1 activity. **(G–I)** Band intensities for pS6K1 and S6K1, and pS6 and S6 in Fig 4E–4G were quantified, and the ratios of pS6K1 to S6K1 and pS6 to S6 are presented as the mean ± SE in **(G)** (related to Fig 4E), **(H)** (related to Fig 4F), **(I)** (related to Fig 4G). Values in control cells stimulated by amino acids for 1 h were set as 1. *P < 0.05. All experiments were repeated 3 times. The data underlying this figure can be found in S1 Data. CRISPR/Cas9, clustered regularly interspaced short palindromic repeat/CRISPR-associated 9; Girdin, girders of actin filaments; mTORC1, mechanistic target of rapamycin complex 1; N.S., not significant; shRNA, short hairpin RNA; siRNA, small interfering RNA; S6K1; S6 kinase beta1; WB, western blot; WT, wild-type.
(TIF)

S2 Fig Girdin and 4F2hc regulate autophagy induced by amino acid depletion. **(A)** 293FT cells transduced with the indicated shRNAs pretreated with or without 200 nM Bafilomycin A1 for 3 h were starved for amino acids (AA–) for the indicated times, followed by WB with the indicated antibodies. Red arrowheads indicate lipidated LC3. The ratio of lipidated to total LC3 is shown in the lower panel. Values in control cells starved for amino acids for 3 h were set as 1. The data underlying this figure can be found in S1 Data. **(B)** Flp-In 293 cells stably expressing the indicated constructs were starved for amino acids (AA–) for the indicated times followed by WB with the indicated antibodies. Red arrowheads indicate lipidated LC3. The ratio of lipidated to total LC3 is shown in the lower panel. Values in control cells starved for amino acids for 2 h were set as 1. The data underlying this figure can be found in S1 Data. **(C, D)** Flp-In 293 cells stably expressing the indicated constructs were transfected with GFP-LC3, followed by starvation for amino acids for 2 h. The cells were then fixed and visualized using confocal microscopy. The fraction of cells (%) with more than 3 GFP-LC3 puncta (100 cells from 3 independent experiments) was quantified in (D). *P < 0.05. The data underlying this figure can be found in S1 Data. GFP, green fluorescent protein; Girdin, girders of actin filaments; LC3, light chain 3; N.S., not significant; shRNA, short hairpin RNA; WB, western blot; 4F2hc, 4F2 heavy chain.
(TIF)

S3 Fig Comprehensive measurement of intracellular amino acids. 293FT cells transfected with indicated siRNA **(A)** or Flp-In 293 cells stably expressing empty vector, Girdin WT, Girdin AA, and 4F2hc **(B)** were starved for amino acids (AA–) for 1 h, stimulated with amino acids for 10 min, and subjected to measurement of intracellular amino acids contents by Agilent 1100 HPLC System. The data underlying this figure can be found in S1 Data. A.U., arbitrary unit; Girdin, girders of actin filaments; siRNA, small interfering RNA; WT, wild-type; 4F2hc, 4F2 heavy chain.
(TIF)

## Abbreviations

Arf1: adenosine diphosphate ribosylation factor-1
Arg: arginine
ATP: adenosine 5′-triphosphate
co-IP: co-immunoprecipitation
CRISPR/Cas9: clustered regularly interspaced short palindromic repeat/CRISPR-associated 9
CT: carboxyl terminal
DG: dentate gyrus
DMEM: Dulbecco’s Modified Eagle Medium
DSS: disuccinimidyl suberate
FBS: fetal bovine serum
FH: Flag-HA epitope
Flag-NT: Flag-tagged Girdin NT domain
GAP: GTPase-activating protein
GEF: guanine nucleotide exchange factor
GFP: green fluorescent protein
Girdin: girders of actin filaments
Gln: glutamine
GPCR: G-protein coupled receptor
GST: glutathione S-transferase
GTPase: guanosine triphosphatase
His-Ub: histidine-tagged Ub
HP: hippocampus
IgG: immunoglobulin G
IPTG: isopropyl beta-d-thiogalactoside
K: lysine
LC3: light chain 3
Leu: leucine
MAPK: mitogen-activated protein kinase
MAPKK: MAPK kinase
MAPKK CA: constitutively active mutant of MAPKK
mLST8: mammalian lethal with SEC13 protein 8
MARCH1/8: membrane-associated RING-CH E3 ubiquitin ligase 1 and 8
Mr: molecular marker
mTOR: mechanistic target of rapamycin
mTORC1: mechanistic target of rapamycin complex 1
N.S.: not significant
NT: amino terminal
OB: olfactory bulb
PRAS40: proline-rich Akt substrate of 40 kDa
pVSV-G: vesicular stomatitis virus G protein
Rf: relative mobility
RMS: rostral migratory stream
RTK: receptor tyrosine kinase
SDS-PAGE: sodium dodecyl-polyacrylamide gel electrophoresis
SE: standard error
sgRNA: single guide RNA
shRNA: short hairpin RNA
siRNA: small interfering RNA
S233: serine at position 233
S237: serine at position 237
S6K1: S6 kinase beta1
TCL: total cell lysate
TSC: tuberous sclerosis
TUBE: tandem-repeated ubiquitin-binding entity
WB: western blot
WT: wild-type
4F2hc: 4F2 heavy chain
