# Supplementary figures and images for: Negative regulation of amino acid signaling by MAPK-regulated 4F2hc/Girdin complex
Source: PLoS Biol. 2018 Mar 14;16(3):e2005090. doi: 10.1371/journal.pbio.2005090 (PMC5868845; doi:10.1371/journal.pbio.2005090)

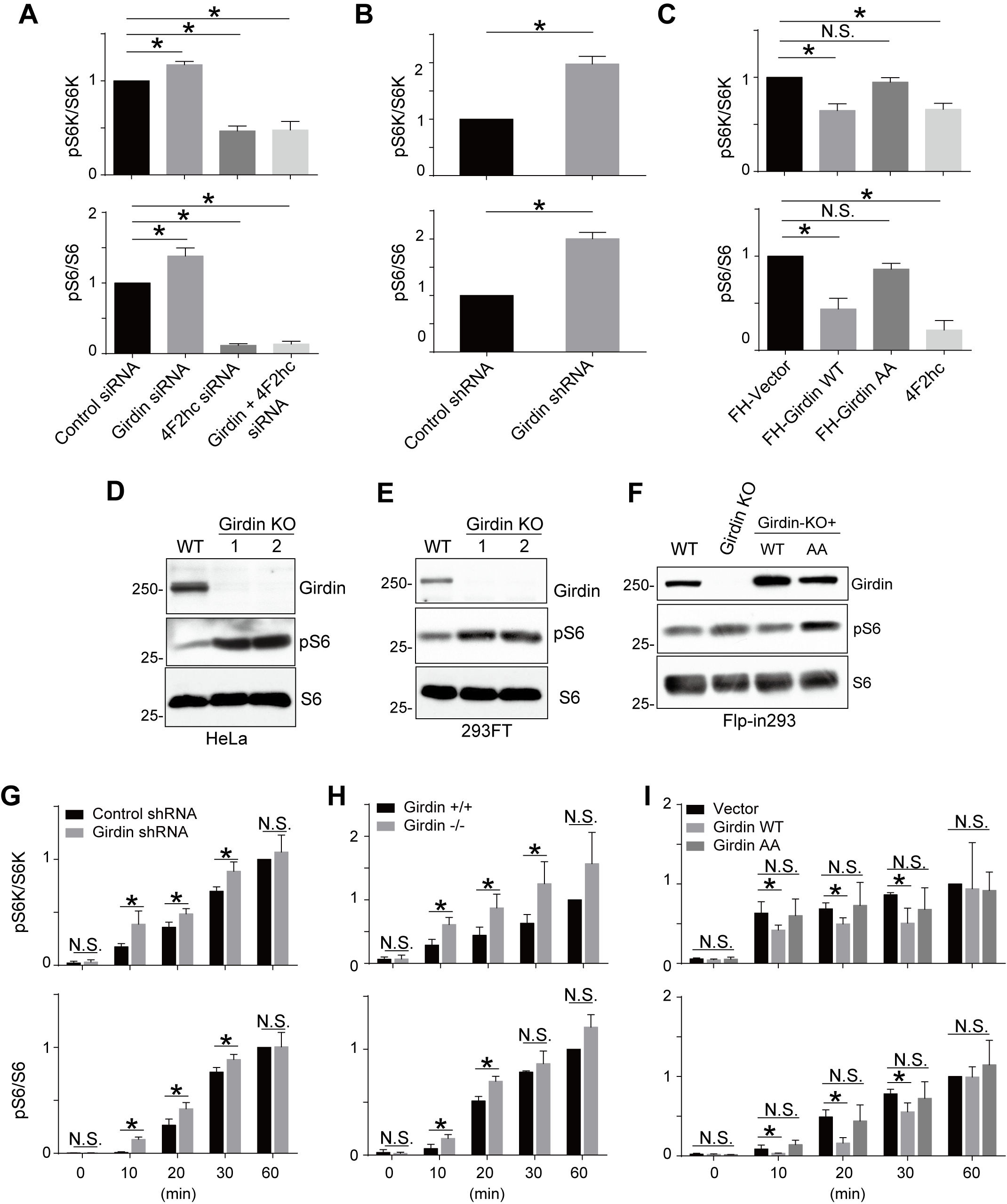

Supplement: S1 Fig — (A–C) Band intensities for pS6K1 and S6K1, and pS6 and S6 in Fig 4A–4C were quantified, and the ratios of pS6K1 to S6K1 and pS6 to S6 are presented as the mean ± SE in (A) (related to Fig 4A), (B) (related to Fig 4B), and (C) (related to Fig 4C). Values in control cells were set as 1. All experiments were repeated 3 times. The data underlying this figure can be found in S1 Data. (D, E) Girdin knockout cells were generated by using the CRISPR/Cas9 system. Lysates from the WT parent cells and Girdin knockout cells were analysed by WB to detect the basal activation level of mTORC1. (F) Girdin WT or AA mutant was re-expressed in Girdin knockout Flp-In 293 cells, followed by detection of basal mTORC1 activity. (G–I) Band intensities for pS6K1 and S6K1, and pS6 and S6 in Fig 4E–4G were quantified, and the ratios of pS6K1 to S6K1 and pS6 to S6 are presented as the mean ± SE in (G) (related to Fig 4E), (H) (related to Fig 4F), (I) (related to Fig 4G). Values in control cells stimulated by amino acids for 1 h were set as 1. *P < 0.05. All experiments were repeated 3 times. The data underlying this figure can be found in S1 Data. CRISPR/Cas9, clustered regularly interspaced short palindromic repeat/CRISPR-associated 9; Girdin, girders of actin filaments; mTORC1, mechanistic target of rapamycin complex 1; N.S., not significant; shRNA, short hairpin RNA; siRNA, small interfering RNA; S6K1; S6 kinase beta1; WB, western blot; WT, wild-type. (TIF) [file pbio.2005090.s003.tif]

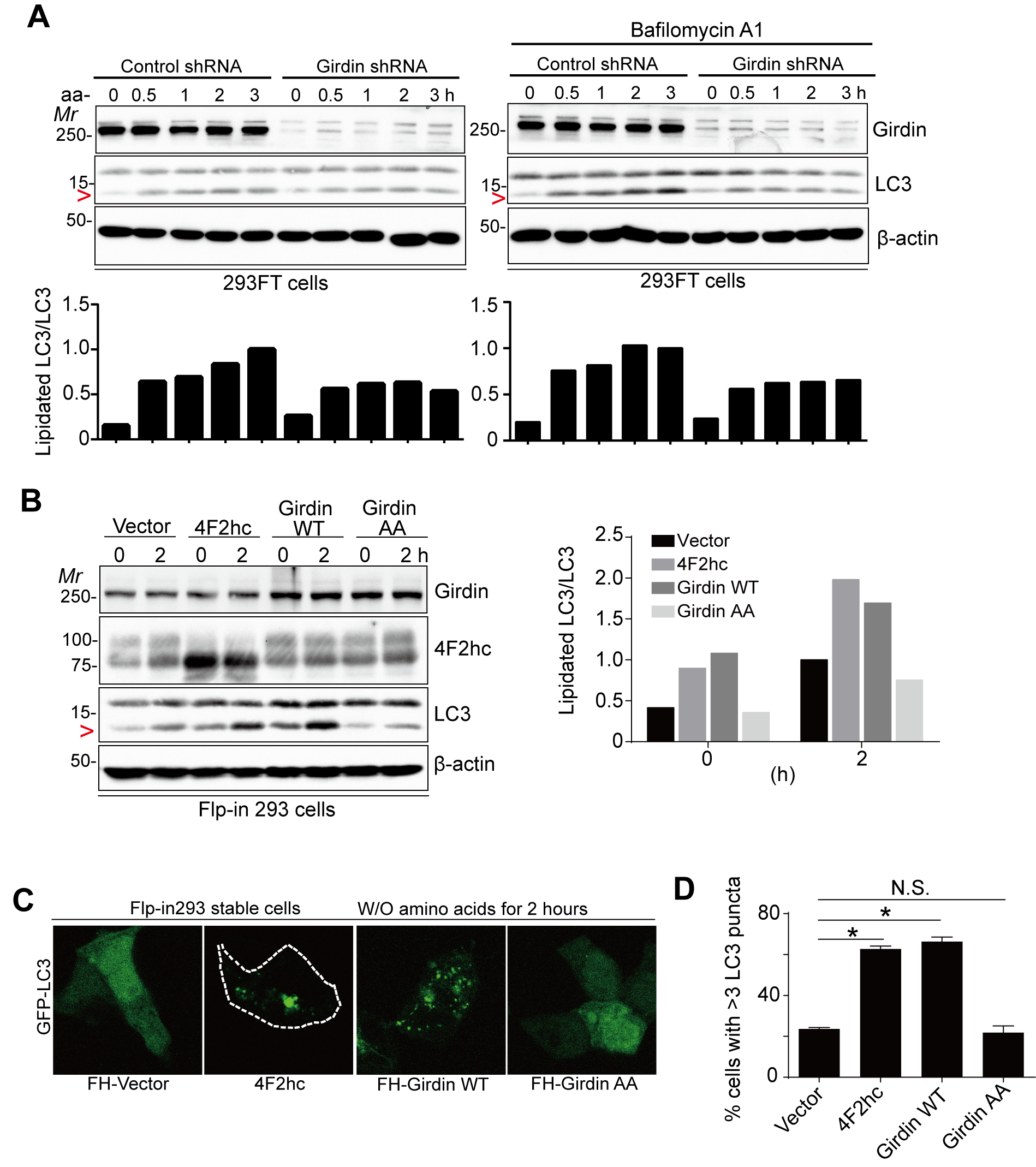

Supplement: S2 Fig — (A) 293FT cells transduced with the indicated shRNAs pretreated with or without 200 nM Bafilomycin A1 for 3 h were starved for amino acids (AA–) for the indicated times, followed by WB with the indicated antibodies. Red arrowheads indicate lipidated LC3. The ratio of lipidated to total LC3 is shown in the lower panel. Values in control cells starved for amino acids for 3 h were set as 1. The data underlying this figure can be found in S1 Data. (B) Flp-In 293 cells stably expressing the indicated constructs were starved for amino acids (AA–) for the indicated times followed by WB with the indicated antibodies. Red arrowheads indicate lipidated LC3. The ratio of lipidated to total LC3 is shown in the lower panel. Values in control cells starved for amino acids for 2 h were set as 1. The data underlying this figure can be found in S1 Data. (C, D) Flp-In 293 cells stably expressing the indicated constructs were transfected with GFP-LC3, followed by starvation for amino acids for 2 h. The cells were then fixed and visualized using confocal microscopy. The fraction of cells (%) with more than 3 GFP-LC3 puncta (100 cells from 3 independent experiments) was quantified in (D). *P < 0.05. The data underlying this figure can be found in S1 Data. GFP, green fluorescent protein; Girdin, girders of actin filaments; LC3, light chain 3; N.S., not significant; shRNA, short hairpin RNA; WB, western blot; 4F2hc, 4F2 heavy chain. (TIF) [file pbio.2005090.s004.tif]

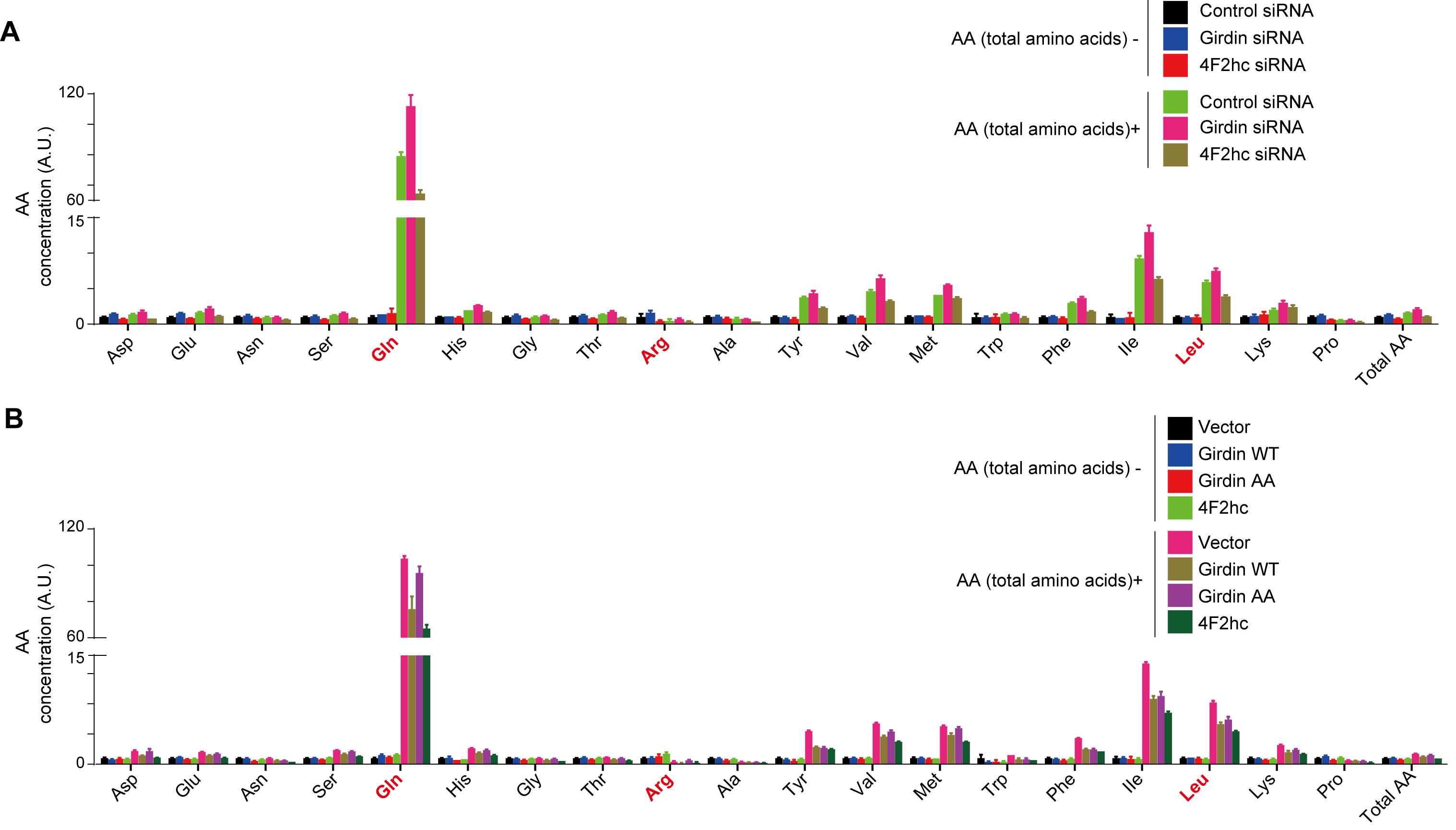

Supplement: S3 Fig — 293FT cells transfected with indicated siRNA (A) or Flp-In 293 cells stably expressing empty vector, Girdin WT, Girdin AA, and 4F2hc (B) were starved for amino acids (AA–) for 1 h, stimulated with amino acids for 10 min, and subjected to measurement of intracellular amino acids contents by Agilent 1100 HPLC System. The data underlying this figure can be found in S1 Data. A.U., arbitrary unit; Girdin, girders of actin filaments; siRNA, small interfering RNA; WT, wild-type; 4F2hc, 4F2 heavy chain. (TIF) [file pbio.2005090.s005.tif]
